# *Aire*-deficient mice provide a model of corneal and lacrimal gland neuropathy in Sjögren's syndrome

**DOI:** 10.1371/journal.pone.0184916

**Published:** 2017-09-19

**Authors:** Feeling Y. Chen, Albert Lee, Shaokui Ge, Sara Nathan, Sarah M. Knox, Nancy A. McNamara

**Affiliations:** 1 Department of Cell & Tissue Biology, University of California San Francisco, San Francisco, California, United States of America; 2 School of Optometry and Vision Science Graduate Program, University of California, Berkeley, California, United States of America; 3 Department of Anatomy, University of California San Francisco, San Francisco, California, United States of America; Xiamen University, CHINA

## Abstract

Sjögren’s syndrome (SS) is a chronic, autoimmune exocrinopathy that leads to severe dryness of the mouth and eyes. Exocrine function is highly regulated by neuronal mechanisms but little is known about the link between chronic inflammation, innervation and altered exocrine function in the diseased eyes and exocrine glands of SS patients. To gain a better understanding of neuronal regulation in the immunopathogenesis of autoimmune exocrinopathy, we profiled a mouse model of spontaneous, autoimmune exocrinopathy that possess key characteristics of peripheral neuropathy experienced by SS patients. Mice deficient in the autoimmune regulator (*Aire*) gene developed spontaneous, CD4+ T cell-mediated exocrinopathy and aqueous-deficient dry eye that were associated with loss of nerves innervating the cornea and lacrimal gland. Changes in innervation and tear secretion were accompanied by increased proliferation of corneal epithelial basal cells, limbal expansion of KRT19-positive progenitor cells, increased vascularization of the peripheral cornea and reduced nerve function in the lacrimal gland. In addition, we found extensive loss of MIST1+ secretory acinar cells in the *Aire* -/- lacrimal gland suggesting that acinar cells are a primary target of the disease, Finally, topical application of ophthalmic steroid effectively restored corneal innervation in *Aire* -/- mice thereby functionally linking nerve loss with local inflammation in the aqueous-deficient dry eye. These data provide important insight regarding the relationship between chronic inflammation and neuropathic changes in autoimmune-mediated dry eye. Peripheral neuropathies characteristic of SS appear to be tightly linked with the underlying immunopathological mechanism and *Aire* -/- mice provide an excellent tool to explore the interplay between SS-associated immunopathology and peripheral neuropathy.

## Introduction

Sjögren’s syndrome (SS) is a complex, chronic, autoimmune disorder that affects as many as four million Americans [1–4]. It primarily affects women (nine women to every man) in the 4^th^ and 5^th^ decade of life and is most prevalent in Caucasians [5]. SS is characterized by focal lymphocyte infiltration of the lacrimal and salivary glands [6,7], which provokes severe and debilitating dryness of the eyes and mouth [8]. Although there is extensive data describing the immunopathology of dry eye disease, current therapies are largely ineffective and none are restorative in nature.

As a systemic autoimmune disorder, SS patients develop multiple neurological manifestations including neuropathies affecting both the autonomic and peripheral nervous system [1,9–11]. Neurological manifestations have long been recognized and the trigeminal nerve is one of the most common cranial nerves affected. In a majority of SS patients [12,13], neurological manifestations are noted to precede sicca symptoms with up to 93% of patients diagnosed with SS after neuropathy symptoms appeared [14]. While the pathogenic mechanisms responsible for most forms of neurological involvement in SS are unknown, there is growing evidence that innervation itself is a negative modulator of inflammation and a positive regulator of progenitor cell-mediated restoration of damaged tissues [15,16]. Changes in corneal sensitivity, nerve morphology and corneal innervation have been observed in SS patients,[17] however, the location of the human lacrimal gland limits the analysis of damage elicited by infiltrating immune cells, and changes in innervation are largely unexplored.

To study the neurological manifestations of SS in the cornea and lacrimal gland, we examined the autoimmune regulator (*Aire*)-deficient mouse model of spontaneous autoimmune exocrinopathy. *Aire* is a putative transcription factor that regulates self-antigen expression in the thymus and thereby prevents autoimmunity by mediating the deletion of potentially self-reactive thymocytes [18]. Previously, we showed that the *Aire* -/- model closely mimics the ocular and glandular-specific clinical manifestations of SS [19,20]. *Aire* -/- mice experience spontaneous organ-specific lymphocytic and mononuclear infiltration of the eye and lacrimal gland that causes a SS-like aqueous-deficient dry eye phenotype where IFN gamma-secreting CD4 T cells serve as the primary effectors and cooperate with local IL-1/IL-1 receptor signaling pathways to induce aqueous tear deficiency and ocular surface disease [21–23].

Here, we present new data demonstrating the spontaneous development of CD4 T cell-mediated neuropathy in both the corneas and lacrimal glands of *Aire* -/- mice. Lymphocytic infiltration of the cornea and lacrimal gland corresponded with notable alterations in functional innervation. Corneal nerve loss occurred throughout the epithelium, but not the stroma, of *Aire* -/- mice and was accompanied by reduced tear secretion, enhanced proliferation, and expansion of corneal KRT19-positive progenitor cells, as well as increased vascularization of the peripheral cornea. Similarly, significant tissue destruction and nerve loss were noted in the lacrimal gland. Innervation of remaining lacrimal gland epithelium was reduced compared to that of WT mice, accompanied by significant reduction of acetycholinesterase (AChE) activity. Finally, ocular surface challenge with topical ophthalmic steroid was sufficient to largely restore corneal innervation, providing strong evidence of a functional link between inflammation and denervation in autoimmune-mediated dry eye disease. Together, our findings show that *Aire* -/- mice provide an excellent model to study the clinical manifestations of neurological disorders associated with SS.

## Materials and methods

### Animal model

Mice were handled in strict accordance with the University of California San Francisco animal welfare guidelines for laboratory and animal care. The protocol was approved by the Institutional Animal Care and Use Committee at the University of California San Francisco (Approval number: AN089075-02). *Aire*-deficient mice were generated by targeted disruption of the murine *Aire* gene (OMIM 240300) as previously described [23], then backcrossed onto the non-obese diabetic (NOD) Lt/J background for more than 10 generations. Genomic DNA isolated from tail clippings was genotyped for the *Aire* mutations by PCR with manufacturer recommended specific primers and their optimized PCR protocols. Both male and female mice were used in the study and were between 8–10 weeks of age when sacrificed.

### Immunohistological and immunofluorescence analysis

Immune cell subtypes were visualized by immunohistochemistry using an antibody specific for CD4 (BD Pharmigen), a donkey anti-rat secondary antibody conjugated to HRP and a DAB staining kit. Briefly, enucleated eyes were embedded in OCT Tissue Tek freezing media, 7μm sections were prepared from tissues using a cryostat (Leica, lzar, Germany) and mounted on SuperFrost Plus slides. Sections were fixed for 10 min in ice cold acetone at −20°C, washed in PBS for 5 min, then blocked with 5% normal goat serum for 1 h at room temperature. After blocking, slides were incubated in 3% hydrogen peroxide to inactivate endogenous peroxidases, then incubated with primary CD4 antibody diluted 1:50 in block overnight at 4°C, washed three times with PBS for 5 min each, and incubated with secondary antibody diluted 1:100 in block for 30 min at room temperature. After three additional washes in PBS for 5 min each, slides were developed with substrate followed by Hematoxylin counterstain. For immunofluorescence, 20 μm sections were incubated overnight at 4°C with one of the following primary antibodies: rabbit anti-TUBB3 (1:500, Covance), rat anti-Ki67 (1:100, Dako), goat anti-cytokeratin-KRT12 (1:100, Santa Cruz), rat anti-KRT19 (1:300; tromaIII, DSHB), rabbit anti-MIST1 (1:1000, gift from Steven Konieczny), goat anti-NKCC1 (1:300, Santa Cruz Biotech), rabbit anti-SEMA3B (1:300, Neuromics), rat anti-Ecadherin (1:300, Life Technologies), rat anti-PECAM (1:300 Milipore). Antibodies were detected using Cy2-, Cy3- or Cy5-conjugated secondary Fab fragment antibodies (Jackson Laboratories), and nuclei were stained with DAPI. Fluorescence was analyzed using a Leica Sp5 microscope and NIH ImageJ software.

The number of CD4+ T cells in the central corneal region was quantified by counting the absolute number of DAB+ brown cells with an associated nucleus labeled using H&E counterstain. The Region of interest (ROI), defined by tissue boundaries using the H&E nuclear staining pattern, was outlined with the freehand tool of ImageJ. The limbal region was defined by ROI and CD4+ T cells in the limbus were quantified by measuring the DAB+ signal implementing Yen’s thresholding methold in ImageJ [24]. Integrated densities within the ROI of the thresholded images were recorded with imageJ. Results are expressed as the average of the mean integrated density per ROI. CD4+ T cells in the lacrimal gland were quantified by measuring the area with DAB+ signal divided by the total area of the whole lacrimal gland and expressed as a percentage of CD4+ T cell infiltration.

Lacrimal gland acinar and ductal populations were labeled for MIST1 and NKCC1, respectively, and imaged by confocal microscopy using a 20μm projection of 1μm confocal sections at 20x objective. The acinar to ductal cell ratio was obtained by dividing the number of MIST1+ cells associated with DAPI-stained nuclei by the number of NKCC1+ cells associated with DAPI-stained nuclei.

Corneal nerves and blood vessels were labeled for beta-3-tubulin (TUBB3) and PECAM, respectively, and imaged by confocal microscopy at 20x using 1μm confocal sections that were combined to produce a 20μm projection. Corneal epithelium or stromal region was outlined by ROI to separate out epithelial and stromal nerve. The area of immunofluorescent signal was quantified by implementing Tsai’s thresholding method (aka Moments) in ImageJ [25]. Integrated density within the ROI of the thresholded image was recorded with imageJ. PECAM imaging was acquired from the limbal region outlined by ROI. A similar thresholding method was used to quantify nerves and blood vessel in the lacrimal gland from a minimum of three 20μm confocal projections per sample using 20x objective. Total Tubb3 or PECAM signal level was normalized by the area of intact epithelial tissue, labeled using Ecadherin, and expressed as normalized nerve density or blood vessel density. For assessing blood vessel dilation, blood vessel area was approximated with an ellipse. The blood vessel diameter was measured as the length of the approximated center line for an approximated ellipse, expressed in micrometer.

Corneal whole mounts from topical steroid-treated *Aire -/-* were labeled for TUBB3 and KRT14, and imaged by confocal microscopy using a 20x objective with 2μm confocal sections that were combined to give a projected thickness of between 60μm and 80μm. Corneal epithelial nerve density was measured as the area of TUBB3 immunofluorescent signal within the KRT14 positive epithelial region, as quantified using Tsai’s thresholding method (Moments) in ImageJ [25]. The ROI was drawn to enclose the KRT14 positive region and integrated density within the ROI of the thresholded image was recorded with imageJ. Nerve density was calculated from a minimum of two 775μm x 775μm microscopic fields per cornea.

Cell proliferation was quantified by counting the number of Ki67+ cells in the central corneal epithelium, limbal epithelium, or lacrimal gland epithelium. To obtain the percentage of proliferating cells per respective region, total cell counts were divided by the number of DAPI-stained nuclei. Corneal epithelial progenitor cells (ePCs) were labeled using an antibody directed against KRT19. The ratio of KRT19 to cornea-specific KRT12 staining was calculated by measuring the relative length of tissue labeled with each marker when assessed limbus-to-limbus beginning at the KRT12 to KRT19 transition zone. The resulting ratio was used as to quantitatively assess the extension of KRT19 into the cornea and, thus, expansion of the progenitor cells compartment.

### Lissamine green staining of the ocular surface

Mice were anesthetized with isoflurane, and 5 μL of lissamine green dye (1%) was applied to the lower conjunctival cul-de-sac. Images were taken using an Olympus Zoom Stereo Microscope (Olympus, Center Valley, PA). Lissamine green staining was scored by dividing the cornea into four quadrants, the extent of staining in each quadrant was classified as Grade 0, no staining; Grade 1, sporadic (<25%); Grade 2, diffuse punctate (25–75%), or Grade 3, coalesced punctate staining (75% or more). The total score was calculated separately for each eye and equaled the sum of all four quadrants ranging from 0 (no staining) to 12 (most severe staining). Scoring was conducted by three masked observers with each data point representing the average score of all observers per mouse cornea.

### Tear secretion measurement

Mice were anesthetized with isoflurane and injected i.p. with 4.5 mg/kg of pilocarpine diluted in saline. Ten minutes later, mice were anesthetized with isoflurane and tear secretion (as indicated by the length of the tear-absorbed region in 15 seconds) was measured using a Zone-Quick phenol red thread (Showa Yakuhin Kako Co. Ltd., Tokyo, Japan).

### Gene expression analysis

Real-time PCR was performed as previously described [26]. Total RNA was collected and purified using RNAaqueous and DNase reagents according to manufacture’s protocol (Ambion, Houston, TX). cDNA prepared using Superscript reagents (BioRad, CA) and SYBR-green qPCR was performed using 5ng of cDNA, with primers designed using *Beacon Designer software*. Target gene expression was normalized using eukaryotic 18s rRNA as an endogenous control. All reactions were performed in triplicate and repeated three times.

### Acetylcholinesterase assay

To assess acinar cell function, the Amplex Red Acetylcholine/Acetylcholinesterase Assay Kit (A12217) (Molecular Probes, Eugene, OR), was used to measure acetylcholinesterase production in three 20μm fresh frozen O.C.T sections of the lacrimal gland.

### Topical steroid treatment

Using a micropipette, 6-week BalbC WT and BalbC *Aire* -/- mice were treated topically with 5 μl of 1.0% prednisolone acetate or vehicle control twice per day for two weeks.

### Statistical analysis

Bar graphs are used to summarize the means and standard errors of each outcome obtained using all data collected from WT and *Aire* -/- mice. Group comparisons were analyzed by different statistical methods depending on the data type. The Wilcoxon sum rank test was used to assess outcomes reported as a ratio or percentage. Log transformed data were analyzed by t test. Corneal CD4 counts were assessed using the Wilcoxon rank sum test. Linear mixed models were applied to control correlation or clustering of the paired two eyes of each mouse for readouts of tear secretion and corneal lissamine score. For all analyses, *P* < 0.05 was considered statistically significant.

## Results

### CD4 T cell-mediated ocular surface damage, lacrimal gland exocrinopathy, and vascular remodeling in the *Aire*-deficient mouse

**Fig 1:**
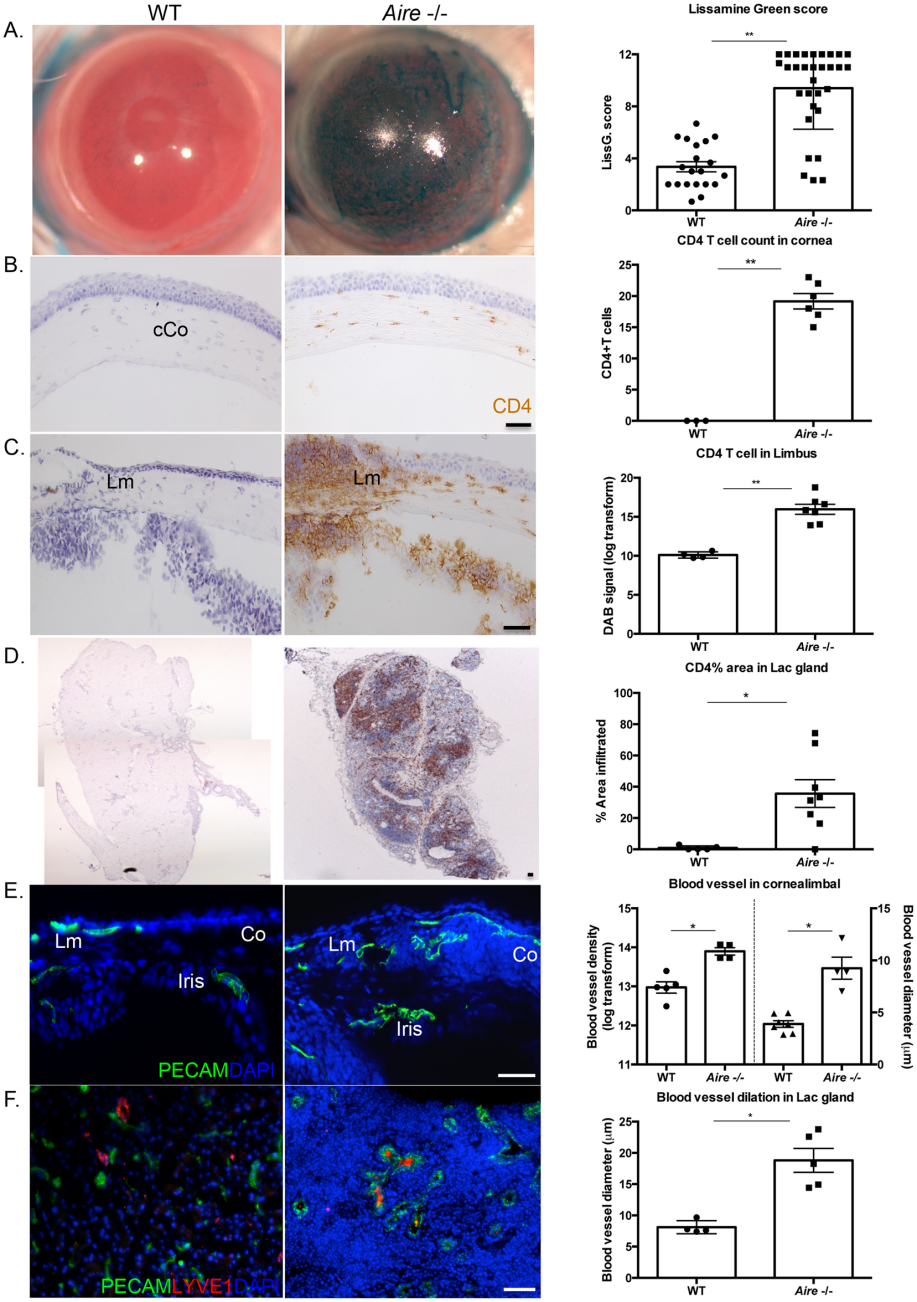
CD4 T cell-mediated inflammation and neovascularization in *Aire* -/- mice. (A) Lissamine green staining indicates significant corneal damage in the *Aire -/-* mice. (B-D) CD4 T cells extensively infiltrate the central cornea (cCo), limbus (Lm), and lacrimal glands of *Aire* -/-. (E) Blood vessels are increased in number and dilated in the corneolimbal region of *Aire* -/-. (F) Lymphatic vessels are closely associated with dilated blood vessels in the *Aire* -/- lacrimal gland. Data are expressed as mean±SEM and are representative of measurements obtained from at least three independent eyes/glands. n ≥ 3 per group. ** *p* < 0.001, *p < 0.05. Scale bar = 50 μm.

*Aire* -/- mice are an established mouse model of autoimmune disease that acquire CD4 T cell-mediated lacrimal gland exocrinopathy, aqueous tear deficiency, and ocular surface damage similar to that observed in SS patients (Fig 1A–1D) [22,27,28]. Compared to wild-type mice, corneas of *Aire* -/- mice exhibited extensive lissamine green uptake (3.24±0.47 vs. 9.55±0.70, *p*<0.001, Fig 1A) and significant lymphocytic infiltration of the central cornea (0.20±0.13 vs. 19.17±0.92, *p*<0.001, Fig 1B) and limbus (10.11±0.12 vs. 15.97±0.51, *p*<0.001, Fig 1C) as early as 6 weeks post-natal. These changes occurred alongside extensive lymphocytic infiltration and foci formation throughout the lacrimal glands (0.44±0.21 vs. 39.68±8.83 percent area per gland, *p* = 0.04, Fig 1D), and significantly reduced tear secretion (18.39±1.22mm vs. 6.09±1.27mm, *p*<0.001). We utilized both male and female mice for this study as both sexes of *Aire* -/- mice exhibited reduced epithelial integrity (lissamine green assessment WT vs. *Aire -/-*: female = 2.89±0.38 vs. 8.62±0.74, *p*<0.0001; male = 3.92±0.39 vs. 7.72±0.67, *p*<0.0001,S1 Fig) and lacrimal gland dysfunction (tear secretion WT vs. *Aire -/-*: female = 16.13±1.49mm vs. 3.08±0.60mm, *p*<0.0001; male = 19.55±1.28mm vs. 13.00±1.77, *p* = 0.01, S1 Fig) compared to their wild type counterparts.

Additional evaluation revealed significant alterations in the pattern and density of blood vessel in the cornea and lacrimal gland of *Aire* -/- mice. Similar to WT corneas, PECAM+ endothelial cells were absent from the central corneas of *Aire* -/- mice but readily apparent in the richly vascularized limbal region (Fig 1E). Blood vessels were significantly dilated in the limbal region of *Aire -/-* corneas (3.89±0.32μm vs. 9.25±1.06μm, *p* = 0.01, Fig 1E) and extended into the peripheral cornea, indicating the early stages of corneal vascularization (12.97±0.11 vs. 13.90±0.06, *p* = 0.02, Fig 1E). Blood vessels in the lacrimal gland of *Aire* -/- mice were similar in number but significantly larger in diameter compared to WT mice (8.35±0.38μm vs. 19.43±1.59μm, *p* = 0.002, Fig 1F). Interestingly, these changes were not accompanied by alterations to the lymphatic network as labeled using LYVE1 (Fig 1F, red). We noted no difference in the pattern or density of lymphatic vessels in the limbal area of the ocular surface and a modest, but not statistically significant, increase in LYVE1+ cells around the ductal regions of the lacrimal gland in *Aire* -/- mice (Fig 1F).

### Increased proliferation and expansion of corneal epithelial progenitor cells (ePCs) in *Aire*-deficient mice

**Fig 2:**
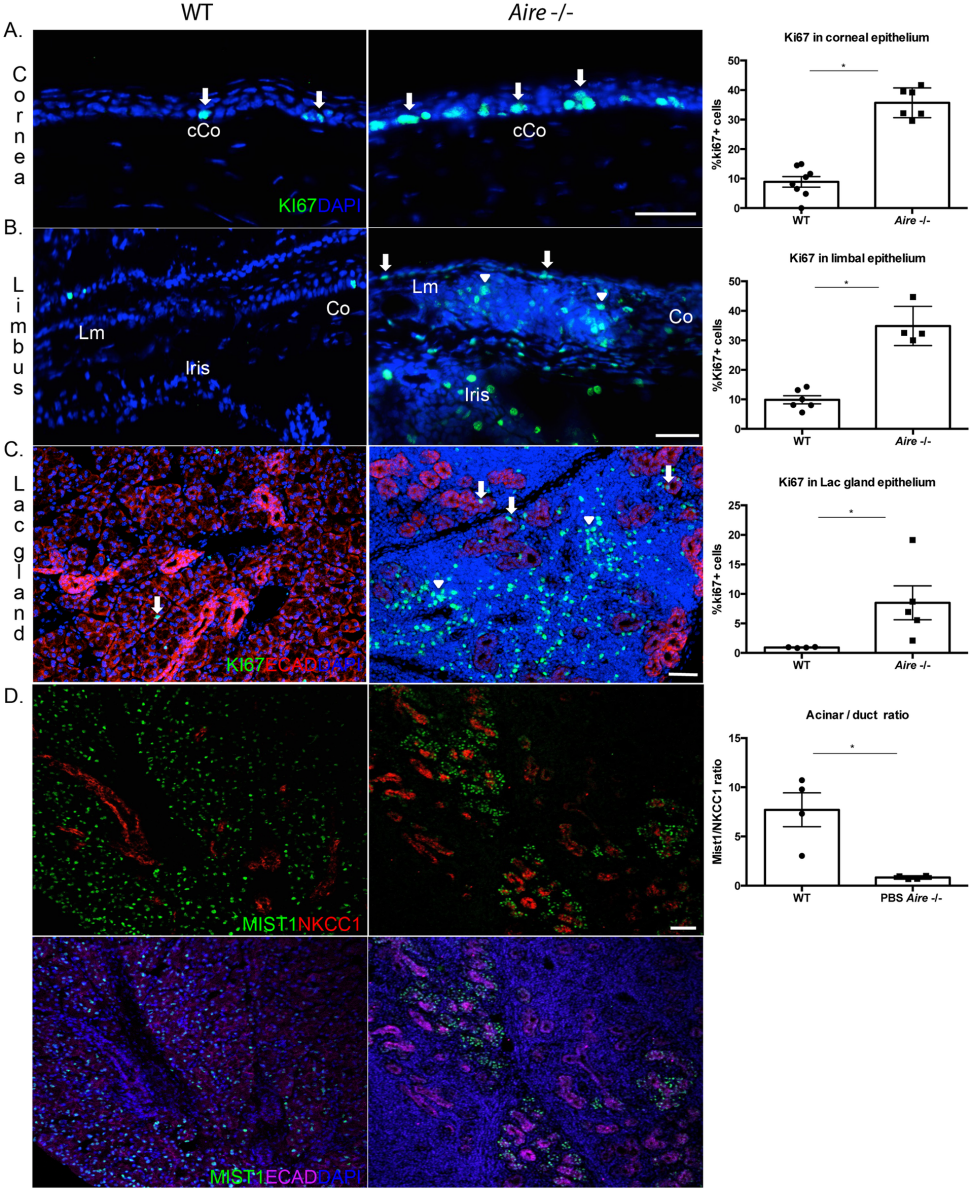
Increased proliferation of ePCs in *Aire* -/- mice. (A-C) Increased proliferation of the Ki67-positive basal epithelial progenitors in the cornea, limbus, and lacrimal gland of *Aire* -/- mice. Arrows indicate Ki67+ proliferating progenitor cells and arrowheads indicate proliferating infiltrating immune cells. (D) The MIST1-positive acinar population but not NKCC1-positive ductal cells are lost in the *Aire* -/- lacrimal gland, as shown in the upper panel; same images are shown with DAPI and Ecadherin in the lower panel. Data in A-D are expressed as means±SEM. Data in D are expressed as a ratio of MIST1- to NKCC1-positive cells. n = 3–6 mice per group. *p < 0.05. Scale bar = 50 μm.

**Fig 3:**
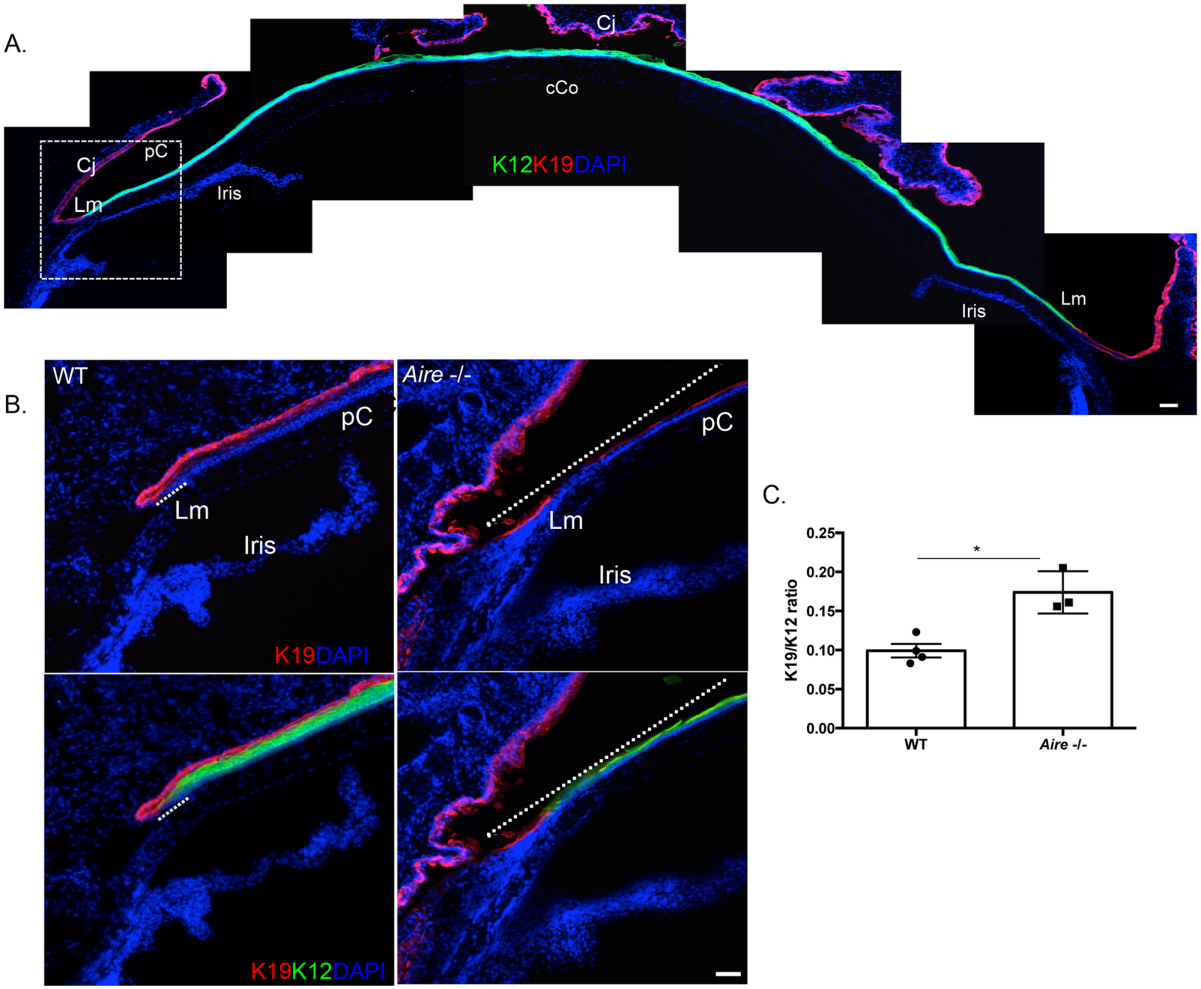
of limbal ePCs into the peripheral cornea of *Aire* -/- mice. **Expansion** (A) Representative composite image of the wild type mouse cornea shows KRT12 expressed throughout the peripheral (pC) and central cornea (cCo), while KRT19 is restricted to the conjunctival (Cj) and limbal (Lm) epithelia. (B) Magnified images of corneal limbal region show extension of KRT19 into the peripheral cornea (pC) of *Aire* -/- mice (upper panel, delineated using a dotted line). Same images shown in the lower panel reveal the length of KRT19-KRT12 overlap (delineated using a dotted line) is significantly less in WT compared to *Aire -/-*. (C) The KRT19/KRT12 ratio was used as a readout to assess extension of KRT19+ ePCs into the peripheral cornea. Data are expressed as means±SEM. n = 3–4 mice per group. *p<0.05. Scale bar = 50 μm.

Proliferation is a natural repair mechanism initiated by ePCs in response to tissue injury. Previously, we showed that CD4 T cell-mediated downregulation of master corneal regulator, PAX6, in *Aire* -/- mice accurately predicted altered differentiation and maturation of corneolimbal epithelial cells [28]. Loss of PAX6 contributed to pathological keratinization of the cornea as indicated by a shift from cornea-specific cytokeratin 12 (KRT12) to epidermal cytokeratin, KRT10. Here, we tested the hypothesis that aberrant differentiation of the corneal epithelium in *Aire* -/- mice is associated with increased proliferation and expansion of ePCs. Using the cell cycle marker, Ki67, to label proliferating cells, we showed a 3-fold increase in the percentage of Ki67-positive cells in both the corneal (9.44±1.50 vs. 35.69±2.06, *p* = 0.004, Fig 2A) and limbal epithelium (10.20±1.61 vs. 34.86±3.32, *p* = 0.03, Fig 2B) of *Aire* -/- mice. Similarly, the ratio of Ki67-positive cells in the epithelial enriched areas of the lacrimal gland was significantly increased in the *Aire* -/- compared to WTs (0.01±0.00 vs. 0.08±0.02, *p* = 0.04, Fig 2C). As we noted extensive destruction of the lacrimal gland and reduced tear secretion, we analyzed the cell types remaining with the hypothesis that acinar cells were likely more impacted than ductal cells. Consistent with this hypothesis, MIST1-positive acinar cells, where MIST1 is a master regulator of the secretory program, were preferentially lost in comparison to the remaining Na-K-Cl cotransporter (NKCC) 1-positive ductal system (7.71±1.21 vs. 0.83±0.06, *p* = 0.03, Fig 2D). Interestingly, increased Ki67 staining in the cornea of *Aire* -/- mice was closely associated with the extension of KRT19-positive limbal ePCs into the peripheral cornea (Fig 3). In WT mice, KRT19 was restricted to the limbal epithelium with a clearly demarcated transition to KRT12-positive corneal cells upon entering the peripheral cornea (Fig 3A). In contrast, KRT19-positive cells extended into the peripheral corneas of *Aire* -/- mice, suggesting expansion of the stem cell compartment and failure of ePCs to differentiate into corneal cells (Fig 3B and 3C, white dotted lines indicate KRT19-postive ePCs).

### Evidence of peripheral neuropathy in the corneas and lacrimal glands of *Aire*-deficient mice

**Fig 4:**
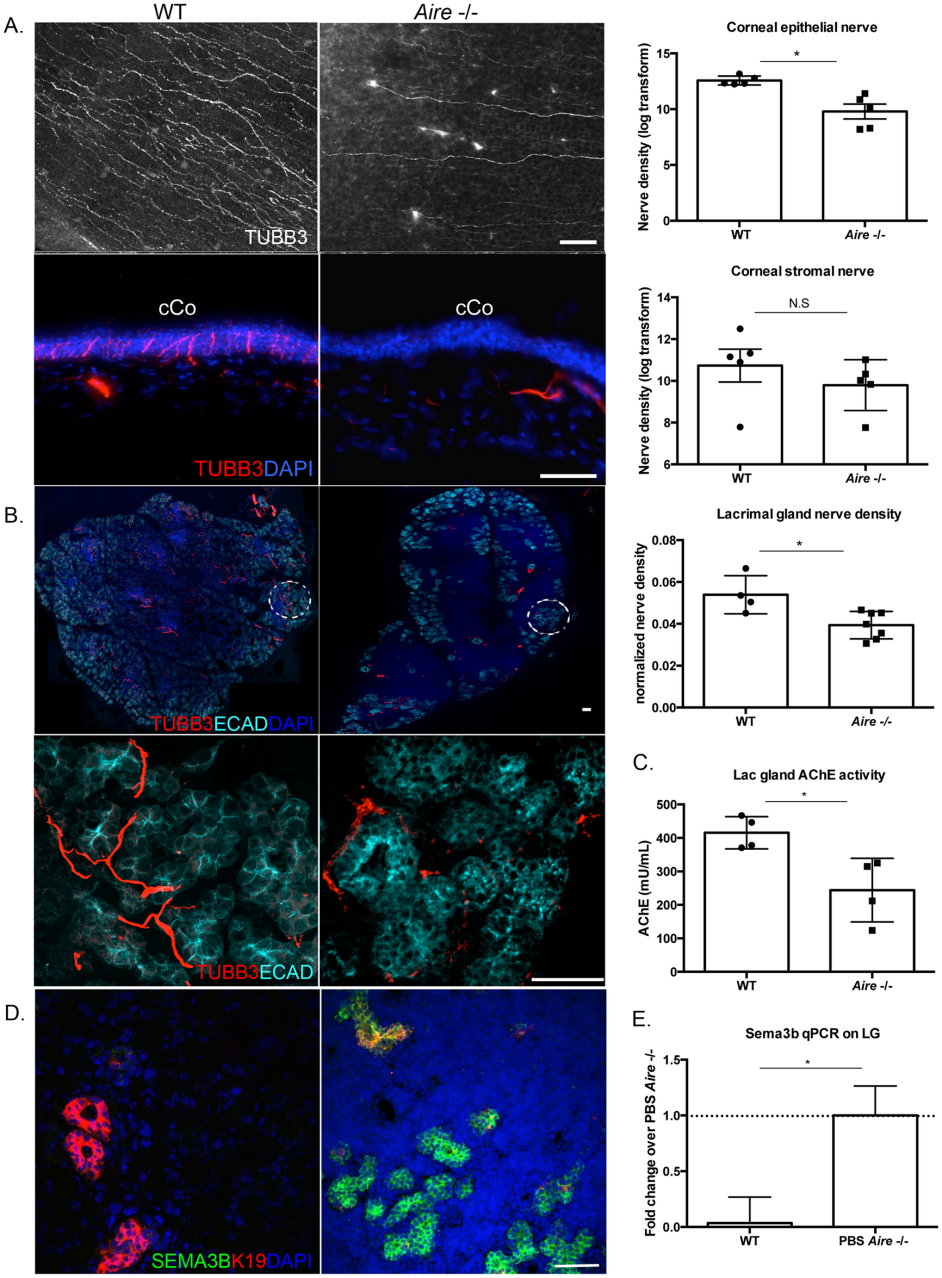
Innervation is reduced in the cornea and lacrimal gland of *Aire* -/- mice. (A) Confocal images of a whole mount cornea labeled for TUBB3 show the *Aire*.*NOD* -/- cornea are devoid of nerves (top left panel). TUBB3 immunostaining of cross-sections of the cornea reveals a similar lack of nerves in the corneal epithelium while stromal nerves are unaffected (lower left panel). (B) Nerves are also lost in the *Aire* -/- lacrimal gland. Global innervation of the lacrimal gland is shown (upper panel) and a zoomed in view of the encircled regions (lower panel) highlight reduced innervation of the remaining epithelium in the *Aire -/-* lacrimal gland. (C) Acetylcholine esterase (AChE) production by the *Aire* -/- lacrimal gland is significantly lower compared to WT lacrimal gland. (D) Representative images of ductal cells in the *Aire* -/- lacrimal gland expressing SEMA3b. (E) Similarly, as shown in the accompanying bar graph, *Sema3b* transcript levels are increased in the *Aire* -/- lacrimal gland. Transcript levels were normalized to the housekeeping gene *Rps29* in wild type controls. Data are expressed as means±SEM. n = 4–7 mice per group. *p<0.05. Scale bar = 50 μm

Many studies have shown loss of corneal innervation in SS-associated dry eye patients [9,17,29]. Corneal nerves are critical for maintaining ocular surface health [29], yet little is known about the mechanism whereby denervation occurs or the extent to which altered innervation is functionally linked to inflammation and/or altered differentiation of the corneolimbal epithelium. Furthermore, due to the location of the lacrimal glands, there has been no analysis of innervation in this organ from patients with SS. We used the pan-neuronal marker tubulin III (TUBB3) combined with confocal microscopy to measure changes in innervation of the cornea and lacrimal gland in the *Aire* -/- mouse (Fig 4). In contrast to WT mice, we found that TUBB3-positive nerves innervating the corneal epithelium were significantly reduced (12.57±0.13 vs. 9.52±0.52, *p* = 0.03, Fig 4A), whereas larger fiber bundles in the stroma and basal plexus were not significantly altered (10.73±0.58 vs. 9.49±0.39, *p* = 0.24, Fig 4A). TUBB3+ nerve fibers innervating the epithelial rich region of the lacrimal gland were also reduced in the *Aire -/-* (0.05±0.005 vs. 0.04±0.002, *p* = 0.04, Fig 4B). To determine the functional capacity of remaining parasympathetic nerves in the *Aire* -/- lacrimal gland (the major nerve subtype eliciting secretion), we measured the activity of acetylcholinesterase (AChE). AChE is required for the termination of neurotransmission at cholinergic synapses through degradation of ACh and is expressed by both nerves and epithelial cells [30]. Consistent with morphological changes noted above, we measured a ~2-fold decrease in AChE activity in the *Aire* -/- lacrimal gland compared to wild type mice (243.96±35.89 vs. 427.89±19.12, *p* = 0.02, Fig 4C). Furthermore, with this alteration in functional innervation, we also found the levels of the axon guidance factor Semaphorin-3B (SEMA3B) protein (Fig 4D) and transcript to be dramatically increased in the *Aire* -/- lacrimal gland relative to WT (1.00±0.26 vs. 0.04±0.23 fold change over *Aire* -/-, *p* = 0.01, Fig 4E) where it was associated with KRT19-positive ductal cells (Fig 4D). Thus, CD4 T-cell induced neuropathy in the corneas and lacrimal glands of *Aire* -/- mice display both structural and functional changes consistent with the human disease.

### Topical anti-inflammatory therapy rescues corneal innervation

**Fig 5:**
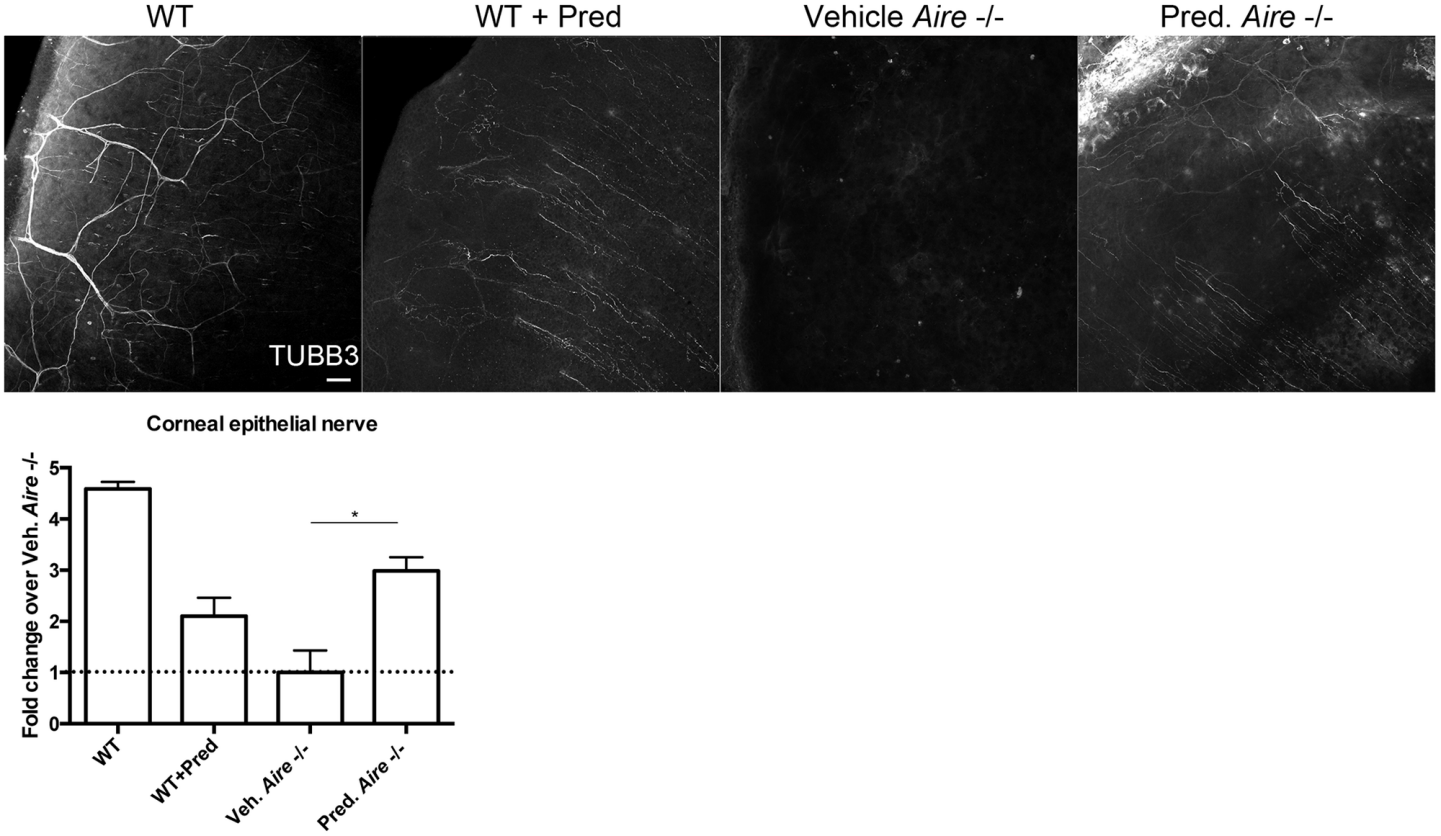
Topical steroid stimulates neurite outgrowth in the *Aire* -/- cornea. Confocal images of whole mount corneas immunolabeled for TUBB3 show that twice-daily topical application of 1% prednisolone acetate significantly increases corneal innervation in *Aire*.*BalbC* -/- mice. Corneal epithelial nerve fiber density in *Aire* -/- mice treated with vehicle (*Aire -/-* + Vehicle) was used as the reference and data expressed as the mean±SEM fold change relative to *Aire -/-* + Vehicle. n = 3–4 mice per group. *p<0.05. Scale bar = 50 μm.

SS-associated dry eye is an immune-mediated disorder and topical anti-inflammatories are the mainstay of therapeutic modalities to manage its clinical signs and symptoms. To test the hypothesis that inflammation plays a key role in mediating corneal nerve damage, we treated *Aire* -/- mice with the topical steroid, 1.0% prednisolone acetate, twice daily for up to two weeks and examined the impact on corneal innervation. Compared to WT mice, corneal innervation was significantly reduced in *Aire* -/- mice and partially restored following treatment with prednisolone (2.98±0.27 vs.1.00±0.43 fold change over *Aire* -/- treated with vehicle, *p* = 0.05, Fig 5). Notably, treatment on the healthy WT cornea with prednisolone caused a mild reduction in corneal innervation, suggesting that chronic use of topical steroid may adversely affect both tissue repair and innervation. These results provide strong evidence linking inflammation and altered innervation of the cornea in aqueous-deficient dry eye and support further exploitation of the *Aire* -/- mouse to examine interactions between the nervous and immune systems in dry eye pathogenesis.

## Discussion

Neurological manifestations of SS have long been recognized to have a substantial negative impact on the ability of target organs to contend with chronic pathological insult. However, a model for this pathological outcome that aligns with the other indicators of the disease has not been reported, thus impeding the development of new therapies. In this study, we sought to investigate neurological features of the cornea and lacrimal gland in the *Aire* -/- mouse model of SS. Clinical manifestations of aqueous-deficient dry eye in *Aire* -/- mice share a number of features with SS patients, including CD4 T cell-mediated exocrinopathy that is accompanied by compromised corneal epithelial integrity, reduced tear secretion, and CD4 T cell infiltration of the ocular tissues and exocrine glands [31]. New findings presented here suggest that changes in clinical characteristics are accompanied by significant alterations in functional innervation, proliferation, and lineage commitment of the diseased corneal epithelium and lacrimal gland [32]. Together, these data strongly support the use of *Aire* -/- mice to study nerve-epithelial-immune cell interactions in SS-associated aqueous-deficient dry eye as a means to define the cellular mechanisms that drive the disease process.

The genetic background of mice continues to be a complicating factor in modeling autoimmune disease. While the spectrum of disease penetrance varies in different strains of *Aire -/-* mice [33], we have shown that both *Aire*.*NOD -/-* and *Aire*.*BalbC -/-* mice develop dry eye and exocrinopathy that mimics the clinical characteristics of SS [20–22,27,28,34,35]. These studies provide evidence that both backgrounds can be utilized to study the impact of dry eye on inflammation, vascularization, and innervation. However, care must still be taken since the time course of disease development differs depending on the background. *Aire*.NOD *-/-* mice develop severe, multi-organ autoimmune disease and have an average life span of approximately 8–9 weeks, whereas *Aire*-/- on the BalbC background have an average life span extending up to ~16 weeks [27]. Accordingly, *Aire*.*NOD* -/- mice develop severe exocrinopathy and dry eye most rapidly between 6–8 weeks, while disease development in BalbC mice occurs more slowly between 6–12 weeks. These latter characteristics have proven *Aire*.*BalbC* -/- to be the preferred background for testing therapeutic interventions [20,35]. Notably, corneal innervation is significantly reduced in *Aire -/-* mice on both the NOD and BalbC backgrounds by 8 weeks, thereby validating both backgrounds for studies of SS-associated immune neuropathy.

While historically considered to function as separate entities, recent work has shown that crosstalk between the immune and nervous systems is essential to maintain immune privilege and peripheral nerve integrity, as well as modulating the repair and regeneration of corneal nerves following tissue injury [31,36]. In contrast, the release of neuropeptides (e.g., CGRP and Substance P) in response injury has been shown to act on resident and infiltrating immune cells to promote neurogenic inflammation and induce symptoms of ocular pain and dryness [37,38]. Immune semaphorins (e.g., SEMA3A, 4A, 4D, 6D, and 7A) are an important group of molecular regulators that link nerve regeneration and inflammatory processes in the cornea by facilitating the interaction between immune cells and sensory nerves to promote axon outgrowth following injury [39]. These interactions are complex, however, and SEMA7A has been shown to have potent angiogenic properties following injection into mouse corneas that were accompanied by enhanced macrophage infiltration and VEGFA release [40]. Thus, the role of neuro-immune crosstalk in the maintenance and repair of inflamed tissues appears to be highly context dependent: an optimal level of inflammation promotes regeneration whereas an insufficient or excessive immune response is ineffective or detrimental to nerve regeneration and repair. While the pathogenic mechanisms responsible for most forms of neurological involvement in SS are unknown, IFN gamma was recently shown to be essential for the development of peripheral neuropathy in *Aire* -/- mice [32]. The mechanism whereby IFN gamma causes peripheral neuropathy remains largely unknown and the impact of IFN gamma on the innervation of other peripheral organs has not been described. Here, we show structural and functional deficiencies in nerves innervating the corneas and lacrimal glands of *Aire* -/- mice. These changes are likely mediated by CD4 T cells, which have been repeatedly identified as key effector cells in the pathogenesis of SS. Both our work and that of others have identified the IFN gamma-secreting, Th1 subset as the predominant player in SS-associated exocrinopathy in *Aire* -/- mice. Furthermore, reducing ocular inflammation with a topical steroid partially restored corneal innervation, suggesting that denervation of the cornea observed in SS patients might be reversible with effective anti-inflammatory therapy. It is important to note, however, that twice daily treatment with topical 1% prednisolone for two weeks caused a modest decrease in corneal nerves in wild type animals, suggesting a potential detrimental connection between topical steroids and corneal nerve supply. Whether chronic use of topical steroids improves or reduces innervation in dry eye patients requires further study.

An essential step toward defining the role of inflammation as a modulator of peripheral neuropathic changes in the pathogenesis of SS-associated dry eye is to first understand the interactions between nerves and epithelial cells in inflamed tissues. Intriguing new studies have shown that corneal epithelial cells support the dense population of innervating corneal nerves by acting as surrogate glial cells that exert molecular and cellular responses similar to those of Schwan cells [41]. Corneal epithelial cells express neuronal markers, including GAP43 and glutamatergic receptors, which facilitate cellular signaling between epithelial cells and nerves during corneal homeostasis and repair. Accordingly, we show that SS-associated dry eye-mediated denervation of the chronically inflamed cornea is accompanied by increased proliferation of corneal epithelial basal cells and altered differentiation of KRT19-positive progenitor cells to their KRT12-positive corneal lineage. These findings are consistent with previous studies demonstrating the importance of innervation in maintaining the proper function of epithelial progenitor cells and suggest that disruption of neuro-epithelial cell interactions in the chronically inflamed eye interferes with differentiation and lineage commitment of the corneal epithelium [42]. Altered innervation may also play a key role in mediating the loss of a pleiotropic transcription factor, paired box gene 6 (PAX6), in both human SS patients and *Aire* -/- mice [28]. PAX6 guides eye morphogenesis during development and was previously shown to provoke altered differentiation of corneal epithelial progenitor cells and the development of sight threatening keratinizing squamous metaplasia of the ocular surface [28]. Further examination of the molecular events underlying functional relationships between corneal epithelial cells and their associated nerves is necessary to fully understand the consequences of prolonged inflammation in SS-associated dry eye disease.

Our study indicates that vascularization of the cornea and the lacrimal gland is increased in the *Aire* -/- mouse. Prior to reaching the epithelial surface, corneal nerves run along blood vessels in the limbus where they help maintain immune tolerance by suppressing inflammation and preventing extension of blood and lymphatic vessels into the peripheral cornea [36]. Nerves supply necessary nutrients to the avascular cornea and under pathological conditions, such as corneal injury and inflammatory disorders, neovascularization and neurogenesis appear to be inversely correlated. In a recent study by Ferrari et al., ablation of the V1 branch of the trigeminal nerve provoked corneal angiogenesis by downregulating the anti-angiogenic factors, pigment epithelium-derived factor (PEDF) and VEGFR3. Conversely, when neovascularization was induced in the cornea, sensory nerves disappeared within areas adjacent to neovessels [43]. Here, we demonstrate a similar phenomenon in a distinct model of corneal inflammation where peripheral vascularization is enhanced in the denervated corneas of *Aire* -/- mice. Such an outcome suggests inflammation alters nerve-blood vessel communication but how this is achieved during dry eye pathogenesis will require further study.

Unlike the labial salivary glands of patients with SS, the location of the lacrimal gland within the orbit limits the ability to perform tissue biopsies. As such, there is little data to determine how the lacrimal gland is impacted by aqueous-deficient dry eye. Our studies in the *Aire* -/- mouse indicate that lacrimal glands undergo extensive damage with acinar cells being preferentially lost to the disease. This finding is similar to that observed in the salivary gland of NOD-scid mice where acinar cells were shown to be selectively diminished [44]. While it is unclear why the ductal system, along with the larger nerve fibers that migrate along them, remain in the face of chronic inflammation, our data suggest that either the nerve fibers are partially resistant to damage or that the ductal system expresses factors during disease progression that help to preserve the nerve supply. In support of this latter notion, we found the secreted axon guidance molecule, SEMA3B, was expressed by lacrimal ductal cells and significantly increased in the *Aire* -/- mice. SEMA3b can bind NRP1 or 2 located on sensory and autonomic nerves and thereby mediate both neuro-attractive and -repulsive functions [45]. Expression of semaphorins is induced by injury in numerous organ systems suggesting these molecules act in repair [46]. Although reduced fluid secretion is thought to be a primary result of lymphocytic infiltration and tissue destruction, lymphocytic infiltration alone may not be sufficient to explain secretory dysfunction in patients with SS since tear reduction has been observed before inflammation in mouse models of the disease [47]. Similarly, saliva production in SS mouse models has been demonstrated to be reduced prior to infiltration of the salivary gland but how this is achieved is not known [44,48]. Our observation of reduced functional innervation to the lacrimal gland highlights a potential source of tissue dysfunction in dry eye patients.

In summary, *Aire* -/- mice provide an exciting model of spontaneous, autoimmune-mediated exocrinopathy that faithfully mimics key clinical features of SS-mediated peripheral neuropathy and immunopathology. Additional investigation of the inter-relationships between the immune and nervous systems, as well as essential structural and functional features that are necessary to maintain neuro-epithelial interactions in the cornea and lacrimal gland, will provide much-needed insight to enhance our fundamental understanding of the peripheral nervous system in the healthy and diseased eye. As we move forward, it will be important to adapt our current conceptual model of dry eye pathogenesis to incorporate the role of corneal nerves and defects in sensory innervation as modulators of the disease process. It is not unlikely that the future of dry eye therapeutics will need to address elements of both the immune and nervous system as we strive to develop restorative therapies that prevent the debilitating signs and symptoms of dry eye disease.

## Supporting information

S1 Fig Male and female *Aire -/-* mice exhibited ocular surface damage and lacrimal gland dysfunction. (A) By 8 weeks, both male and female *Aire -/-* had significantly higher corneal lissamine green score compared to their WT counterparts. (B) Both sexes of *Aire -/-* mice showed substantial reduction in tear secretion compared to the WT counterparts. Data are expressed as mean±SEM and are representative of measurements obtained from at least eight independent eyes. n ≥ 4 per group. ** *p* < 0.001, *p < 0.05.
(TIF)
