# Supplementary figures and images for: Aire-deficient mice provide a model of corneal and lacrimal gland neuropathy in Sjögren's syndrome
Source: PLoS One. 2017 Sep 19;12(9):e0184916. doi: 10.1371/journal.pone.0184916 (PMC5605119; doi:10.1371/journal.pone.0184916)

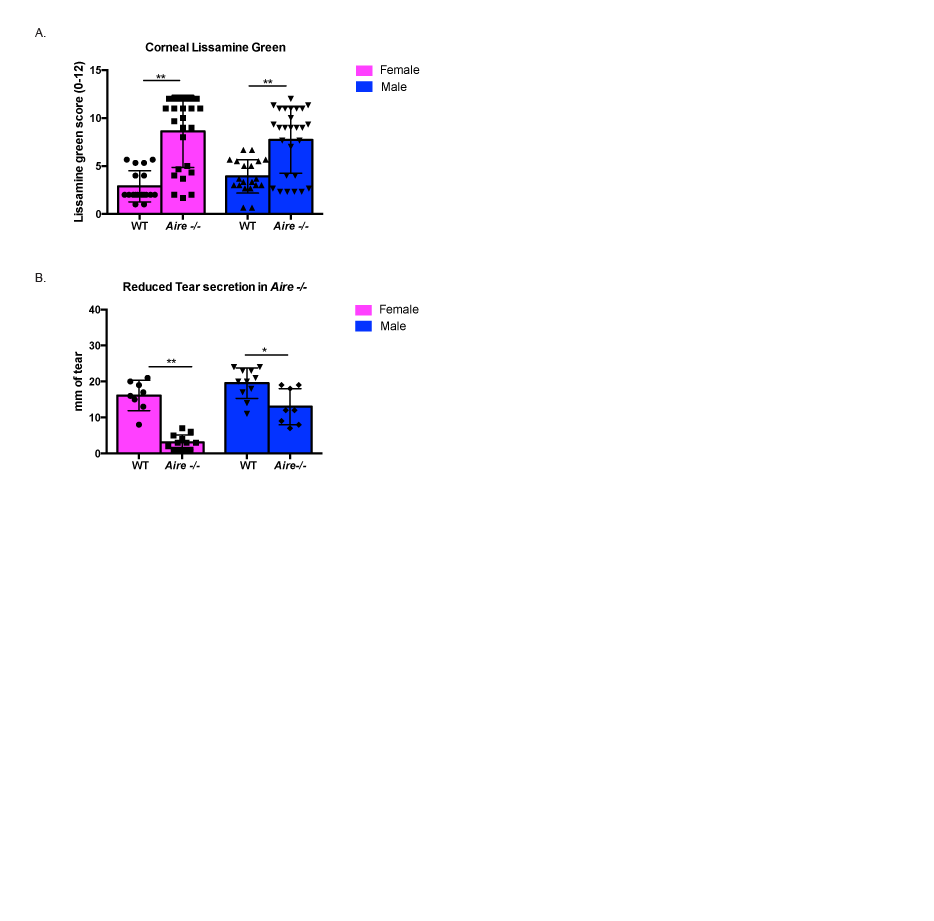

Supplement: S1 Fig — (A) By 8 weeks, both male and female Aire -/- had significantly higher corneal lissamine green score compared to their WT counterparts. (B) Both sexes of Aire -/- mice showed substantial reduction in tear secretion compared to the WT counterparts. Data are expressed as mean±SEM and are representative of measurements obtained from at least eight independent eyes. n ≥ 4 per group. ** p < 0.001, *p < 0.05. (TIF) [file pone.0184916.s001.tif]
